# Telescopic synthesis of cellulose nanofibrils with a stable dispersion of Fe(0) nanoparticles for synergistic removal of 5-fluorouracil

**DOI:** 10.1038/s41598-019-48274-2

**Published:** 2019-08-12

**Authors:** Mohd Shaiful Sajab, Denesh Mohan, Jude Santanaraj, Chin Hua Chia, Hatika Kaco, Shuhaida Harun, Nur Hidayatul Nazirah Kamarudin

**Affiliations:** 1 Research Center for Sustainable Process Technology (CESPRO), Faculty of Engineering and Built Environment, Universiti Kebangsaan Malaysia, 43600 Bangi, Selangor Malaysia; 2 Chemical Engineering Programme, Faculty of Engineering and Built Environment, Universiti Kebangsaan Malaysia, 43600 Bangi, Selangor Malaysia; 3 School of Applied Physic, Faculty of Science and Technology, Universiti Kebangsaan Malaysia, 43600 Bangi, Selangor Malaysia; 4 Kolej GENIUS Insan, Universiti Sains Islam Malaysia, Bandar Baru Nilai, 71800 Nilai, Negeri Sembilan Malaysia

**Keywords:** Nanoparticles, Pollution remediation

## Abstract

The recognition of cellulose nanofibrils (CNF) in the past years as a high prospect material has been prominent, but the impractical cellulose extraction method from biomass remained as a technological barrier for industrial practice. In this study, the telescopic approach on the fractionation of lignin and cellulose was performed by organosolv extraction and catalytic oxidation from oil palm empty fruit bunch fibers. The integration of these techniques managed to synthesize CNF in a short time. Aside from the size, the zeta potential of CNF was measured at −41.9 mV, which allow higher stability of the cellulose in water suspension. The stability of CNF facilitated a better dispersion of Fe(0) nanoparticles with the average diameter size of 52.3–73.24 nm through the formulation of CNF/Fe(0). The total uptake capacity of CNF towards 5-fluorouracil was calculated at 0.123 mg/g. While the synergistic reactions of adsorption-oxidation were significantly improved the removal efficacy three to four times greater even at a high concentration of 5-fluorouracil. Alternatively, the sludge generation after the oxidation reaction was completely managed by the encapsulation of Fe(0) nanoparticles in regenerated cellulose.

## Introduction

In 2017, the area of oil palm plantations in Malaysia has increased to 5.8 million hectares. In further detail, the average fresh fruit bunch production was 15.9 tonnes per hectare^1^. Despite the annual increment in oil palm production (and hence, oil palm biomass), these by-products have been underutilized^2^. Since the primary components of the lignocellulosic biomass of oil palm predominantly contain cellulose, hemicelluloses, and lignin, the aforementioned form of biomass possesses desirable properties for the formation of biodegradable polymers. For example, cellulose is a carbohydrate polymer comprising repetitive units of β-D-glucopyranose joined by β-1,4-glycosidic bonds. Cellulose has been utilized on a wide variety of applications due to its characteristics on renewable, low price, high availability and good mechanical properties^3^. While the conversion of cellulose to regenerated cellulose providing more flexibility in the microstructure and physical form of the polymer^4^.

Despite the importance of cellulose in next-generation polymers, the industry is yet to fully equip itself with suitable technologies for the large-scale fractionation of nanocellulose from underutilized biomass. Evidently, impractical methods of cellulose extraction from biomass remain to be a technological barrier in this industry. In cellulose isolation, pretreatment and chemical extraction are the typical processes by which lignin and hemicellulose of lignocellulosic compounds are deconstructed into soluble ones^5,6^. Furthermore, repetitive pulping and bleaching are required to obtain cellulose of high purity. As such, the abovementioned series of processes do not only give poor cellulose yields; they also produce large amounts of effluents (black liquor). In fact, some of the pretreatment methods employ chlorine-based chemicals as well as generate toxic substances and chlorine radicals which endanger the environment. However, with proper isolation and purification techniques, lignin can be converted into polymers of high quality^7^.

Advanced organosolvation (organosolv) fractionating technologies for lignocellulosic biomass are able to increase the efficiency of the recovery of individual lignocellulosic components^8^. Specifically, the chemicals extracted via this process can be easily recovered through distillation, hence makes it cost-effective and eco-friendly^9^. As an alternative, catalytic oxidation with hydrogen peroxide (H_2_O_2_) as the oxidation agent improves the efficiency of delignification^10^. Ergo, the chemical and electrocatalytic activities during the depolymerization of lignocellulosic materials augment cellulose recovery and facilitate the development of more promising substances^10–13^. Meanwhile, Fenton oxidation deconstructs lignin polymers and can further depolymerize cellulosic as well as hemicellulosic substances under mild conditions^14–16^.

On another matter, the conversion of cellulose into nanocellulosic cellulose nanocrystals (CNC) and cellulose nanofibrils (CNF) reportedly gave rise to promising materials for a diversity of applications, e.g. polymer reinforcers, substance thickeners, electronic substrates, water-remediating agents, etc^17^. While CNF is regarded as a potential rapid nanocomposite adsorbent, the synergy between its adsorptive and multifunctional properties has yet to be translated into a more environmental-friendly water remediation^18^. The catalytic activity such zero valent iron (Fe(0)) nanoparticles has been prominent Fenton’s reagent for water remediation process^19^. Basically, Fenton’s reagent will be associated with H_2_O_2_ in the propagation reaction of hydroxyl radicals (•OH). Despite the strong degree in organic degradation, the complete reaction of Fenton oxidation was promoted a sludge generation, which will indirectly be composed of secondary solid waste and acquire additional treatment process^20,21^. Besides, the formation of Fe(0) nanoparticles was highly unstable, segregated and easily oxidized^22–24^. As an alternative, the composition of Fenton to Fenton-like (Fe(II), Fe_3_O_4_, Fe(0)) reagents with the large surface substance as a template has been provided a better uniformity, highly stable and stimulated synergistic adsorption-oxidation reaction^25–28^. The immobilization of the heterogeneous Fenton’s reagent was mostly involved with highly porous materials varies from clays, zeolites, silica and carbon-based materials^29^. Nevertheless, the immobilization of Fenton’s reagent on a supporting medium generates a more stable catalyst, in particular, the highly-reactive catalyst of zero valent iron (Fe(0))^30^. The large surface area of graphene oxide (GO) for example, comprises with a great number of reactive oxygen functional groups (hydroxyl, epoxy, carbonyl and carboxyl) to provide an impeccable technique for the higher distribution of nanoparticles and effective filler for cellulose-based materials^18,31^.

Recently, notwithstanding the wide range of compounds present in industrial effluents, the cytostatic drug effluents released by hospitals can be traced in sewage treatment plants and are raising concerns over their potential public health threats. It is difficult for antimetabolites like 5-fluorouracil (5-FU) to be biodegraded completely^32,33^. Also, the removal of highly polar contaminants of 5-FU via adsorptive techniques constitutes an uphill task, even for commercial activated carbons^34^. The capacity of catalysis in water remediation was essential in removing persistent contaminants which are strongly resistant chemical to biodegradation^35,36^. Accordingly, the high efficacy of Fenton oxidation makes it a practical approach to the removal of pharmaceutical effluents, apart from the generation of iron sludge^22,37,38^.

Thus, in this study, in order to reduce the loss of cellulose and generation of chemical waste during the pretreatment, cellulose-isolation, and defibrillation of lignocellulosic substances, a telescopic approach has been developed with due attention to the processes that precede and follow the complex fractionation of lignocellulosic fibers. The telescopic synthesis of CNF entailed a series of lignin-extraction, catalytic-oxidation, and mechanical-shearing processes. Additionally, the excess Fe(II) ions generated during catalytic oxidation was used for the *in situ* synthesis of Fe(0) nanoparticles and make use for the functionalization of CNF in water remediation. Individual adsorption and catalytic degradation studies were performed to evaluate the synergistic removal of 5-FU by CNF and Fe(0). Furthermore, the stability of the colloidal Fe(0) nanoparticles were examined in terms of oxidization, in comparison with the formulations of Fe(0) in CNF/Fe(0) and encapsulated in regenerated cellulose in water suspension.

## Materials and Methods

### Materials

Oil palm empty fruit bunch (EFB) fibers were procured from Szetech Engineering Sdn Bhd (Selangor, Malaysia). These were milled and sieved to obtain fibers of diameters 106 to 500 µm. Fractionation of the oil palm EFB pulp was performed using 90% formic acid (Merck) and 30% hydrogen peroxide (Merck). Lignin content was determined using 98% sulfuric acid (Merck). With regards to the preparation of Fenton’s reagent and Fe(0) nanoparticles, a stock solution of Fe(II) was prepared from Fe_2_SO_4_ (Merck), with a small amount of 1 M hydrochloric acid (Merck) added to prevent sedimentation (~0.1 mL). The reducing agent here was sodium borohydride (Merck). Stock solutions of 5-fluorouracil (Merck) were prepared with reference to a standard calibration curve generated by a UV-Vis spectrophotometer equipped with a sipper and peltier (Spectrum SP-UV 300SRB) at a λ_max_ of 265 nm. Sodium acetate (Merck) was the buffer solution for this process. Graphene oxide (GO) was synthesized using graphite flakes (Ashbury, Inc. USA), phosphoric acid, 85% (Merck), potassium permanganate, 99.9% (Merck), hydrogen peroxide, 30% (Merck).

### Delignification and cellulose isolation

During fractionation of lignocellulosic fibers, lignin extraction was carried out using EFB and 90% formic acid (FA) (weight ratio 1:30, 90% w/v) at 90 °C for 2 h. The chemicals were placed in three-necked, flat-bottomed flasks equipped with condensers. Temperature and stirring speed were controlled using a digital hotplate magnetic stirrer (MSH-20D, Daihan Scientific, Korea). The supernatant and pulp were separated by a modified vacuum filter with an extended hose filter.

Following removal of the supernatant, the treated EFB fibers (cellulosic pulp fraction) was subjected to continuous purification via catalytic oxidation at a low concentration of H_2_O_2_ (1–6% w/v) in the presence of by Fe(II) (2–14 mg/L) at 90 °C for 24 h. The degradation kinetics of lignin, or the catalysis of the EFB fibers, was monitored closely at various time intervals over 24 h. The extracted cellulose was washed with deionized water and kept in a refrigerator (~4 °C) until further use. Throughout fractionation, cellulose purity and lignin yield were monitored by means of compositional analysis (TAPPI T222 os-06 (2006) standard) and further characterized via chemical analyses.

### Cellulose defibrillation and Fe(0) formation

The isolated cellulose was subjected to mechanical shearing to generate CNF EFB^17^. Specifically, the cellulose was mixed with deionized water (0.7 wt%) and then shredded in a high-speed blender at 37,000 rpm (Vitamix 5200, Vitamix, Ohio, United States) for up to 30 min with a control cycle. The temperature during the entire defibrillation process was kept constant at 70 °C. Meanwhile, *ex situ* synthesis of Fe(0) was carried out by mixing iron (of 1:2 control weight ratio) and a reducing agent – NaBH_4_ – at 200 rpm for 1 h. This acted as a control sample. On the other hand, the *in situ* generation of Fe(0) from the CNF EFB suspension entailed the mixing of 10 wt.% of Fe(II) ions on the CNF EFB plus 1:2 weight ratio of iron and NaBH_4_ with stirring for 1 h. All prepared samples were further sonicated and kept in suspension form until further use.

### Preparation of cellulose beads encapsulated GO/Fe(0)

Cellulose solution was prepared by dissolving 3 g of CNF in NaOH/urea (7:12 weight ratio) precooled solvent at −13 °C and stirred for 5 min to obtain a clear cellulose solution. The obtained cellulose solution was centrifuged at 14,500 rpm for 3 min to remove the bubbles in the cellulose solution. While GO solution was prepared according to Hummers’ method^39^. Briefly, oxidation process was carried out with a mixture of H_2_SO_4_ (360 mL), H_3_PO_4_ (40 mL), KMnO_4_ (18 g) and graphite flakes (3 g) was stirred for three days. Subsequently, the oxidation reaction was suspended with adequate amounts of ice (400 mL) and H_2_O_2_ (27 mL). The solution was washed with HCl (1 M) up to three times and proceed with deionized water ten times until the pH of the solution reached ~pH 3. The *in situ* formation of Fe(0) nanoparticles on the GO was done by the deposition of Fe(II) ion on the GO. Concisely, 10 wt.% of Fe(0) was prepared with the mixture of Fe(II):NaBH_4_ at 2:1 (weight ratio) in the GO suspension (6.2 g/L) and continuously stirred for 1 h. Then, the suspension solution was further sonicated for 30 min, and kept until further use.

The formation of regenerated cellulose beads can be formed by continuously dropping cellulose solution through a syringe nozzle in the coagulation bath (5% H_2_SO_4_). The formation of encapsulated GO/Fe(0) can be formed by continuously dropping GO/Fe(0) solution into cellulose solution and hold for approximately five minutes or the formation of regenerated cellulose occurs on the top layers of GO/Fe(0). The beads were washed several times with distilled water to remove the excess of unreacted cellulose solution. Finally, the beads was freeze-dried for 24 h and kept in the desiccators until further use.

### Adsorption-oxidation on 5-FU removal

The adsorption capacity was determined by mixing 0.1 g of the adsorbent in 30 mL of 5-FU (2–10 mg/L) for 4 h at 200 rpm and room temperature. An aliquot of the solution (~0.1 mL) was withdrawn and subjected to UV-Vis spectrophotometry at various time intervals. On another note, the oxidation was performed using a similar experimental setup, with the addition of Fe(0) nanoparticles. The integration of both techniques (adsorption-oxidation), was carried out for composited materials of CNF/Fe(0) and encapsulated GO/Fe(0) cellulose beads. The efficiency of the adsorption, oxidation and adsorption-oxidation were analyzed according to the following Eqs (1) and (2):

1 $${q}_{t}=\frac{({C}_{0}-{C}_{{\rm{t}}})V}{m}$$

2 $$ \% \,{\rm{of}}\,{\rm{adsorbate}}\,{\rm{removal}}=\frac{({C}_{0}-{C}_{{\rm{t}}})}{{C}_{0}}\times \mathrm{100} \% $$

where, *C*_0_, *C*_t_ and *C*_e_ are the initial concentration, the concentration of 5-FU at the time, *t*, and concentration at the equilibrium (mg/L), *m* and *V* is the mass of adsorbent (g) and volume of adsorbate solution (L).

### Characterization

The pre- and post-processing morphologies of the sample were observed using a variable-pressure scanning electron microscope (VPSEM) (Merlin Compact, Zeiss Pvt Ltd., Oberkochen, Germany), while the Fe(II) residues in the final sample identified using energy-dispersive analysis spectroscopy (EDS). The concentration of Fe(II) ions has been measured using atomic absorption spectroscopy (AAS) (Perkin Elmer AAnalyst 800). The micrographs of CNF EFB, Fe(0) and GO were obtained via transmission electron microscopy (TEM) (CM 12 Philips, Eindhoven, Netherlands). Some 0.01 wt% of each of the two nanoparticles were mixed with ethanol and stained with 3 wt% of uranyl acetate. The crystalline regions of the samples were studied using an X-ray diffractometer (XRD) (Bruker D8 Advance, Bruker, Billerica MA, USA). Fourier-transform infrared spectroscopy (FTIR) (Bruker, Billerica MA, USA) was then performed to characterize their functional groups at a resolution of 1 cm^−1^ in the range of 4 000 to 650 cm^−1^. Additionally, the said samples had their zeta potentials determined using a Zetasizer (Malvern, Worcestershire, UK).

## Results and Discussion

### Optimization of lignocellulosic delignification

**Figure 1:**
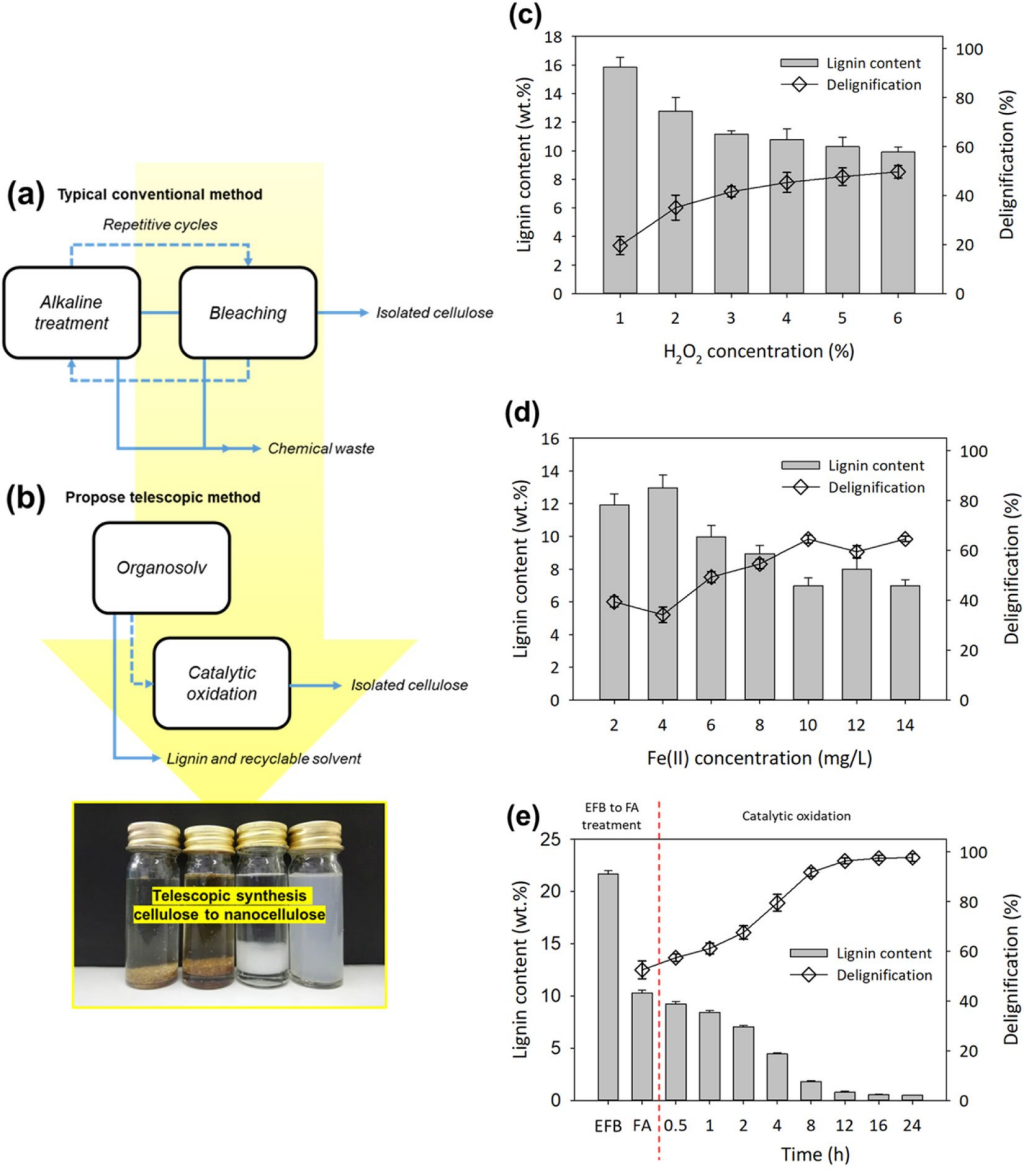
The flow cycles of cellulose isolation through (**a**) conventional method (**b**) integration method and the effect of lignocellulosic delignification on the control parameters of (**c**) H_2_O_2_ concentration, (**d**) Fe(II) dosage on catalytic oxidation and (**e**) delignification kinetics of the integration organosolv and catalytic oxidation.

Typically, lignocellulosic delignification – which produces cellulose to nanocellulose – entails the disruption of cellulose, hemicellulose, and lignin using a combination of mechanical and chemical pulping, steam explosion, hydrolysis, as well as alkaline and bleaching processes^40,41^. A similar technique has been reported in our previous study, whereby alkaline-bleaching was performed repetitively up to six times until the desired purity of cellulose was obtained^17^ (see Fig. 1a). Besides, at the end of every stage, thorough washing was required to neutralize the sample. Accordingly, the proposed telescopic method (in which FA was the organic solvent) might be able to maintain the continuity of the subsequent processes of delignification as well as remove undissolved lignin residues via catalytic oxidation, or Fenton oxidation (see Fig. 1b). From there, downstream sample-washing and -neutralization would be unnecessary as the delignified FA residues on the EFB fibers were sufficiently good for the fractionation of lignocellulosic fibers^8^.

In the preliminary study, the control FA, H_2_O_2_, and Fe(II) concentrations for lignocellulosic delignification have been evaluated in advance to optimize the conditions for lignin and cellulose fractionation. One of the initial parameters comprised untreated EFB fibers at 21.7 wt% of initial lignin content; this was the sample reference. Delignification in the presence of 90% FA gave the highest activity such that the lignin content was reduced to 10.29 wt%. Evidently, a higher concentration of FA is expected to improve lignin dissolution through acidic cleavage of the ethers in the lignin macromolecules^42,43^. However, in organosolv extraction alone, a repetitive cycle is required to achieve a significantly higher ratio of FA. Since organosolv is still incapable of isolating cellulose with a single cycle of reaction, telescopic catalytic oxidation has been performed to remove the remaining insoluble lignin in the EFB fibers.

To enhance the purity of the isolated cellulose, oxidation (bleaching) of the remaining insoluble lignin appears to be a viable approach. Unlike other techniques (catalytic cracking, hydrolysis, reduction, etc.), lignin depolymerization via oxidation is favored as it does not disrupt the cellulosic composition^44^. Accordingly, the optimized parameter of untreated EFB fiber oxidation consisted of H_2_O_2_ in the range of 1 to 6% (see Fig. 1c). Evidently, 3% of H_2_O_2_ effectively reduced the lignin content by up to 41.6%. Meanwhile, delignification of lignocellulose using higher concentrations of H_2_O_2_ yielded a narrow range of 9.9–10.8 wt% of insoluble lignin residues in the EFB fibers. This, by the way, was consistent with the results of our previous study^16^.

Catalytic oxidation (Fenton oxidation) reactions were carried out using different amounts of Fe(II) (see Fig. 1d). It was then discovered that the optimum level of Fe(II) concentration was 2 to 14 mg/L. Evidently, the raise in Fe(II) increased the extent of delignification, generation of iron oxide residues. Low concentrations of Fe(II) consistently doubled the efficiency of oxidation, giving rise to a maximum of ~64.5% of delignification^14^. The hydroxyl radicals generated during Fenton oxidation were selectively targeted towards lignin, which turned out to be an efficient method for the delignification of lignocellulose^16^. Evidently, at moderate concentrations of H_2_O_2_ and Fe(II), the changes in the cellulosic structure were insignificant^45^. As a side note, the concentrations of the catalyst and oxidant were capped at 10 mg/L of Fe(II) as further increments in the availability of hydroxyl radicals could further depolymerize the cellulosic composition and weaken the structure of the fibers^11^.

With respect to the best conditions for lignocellulose delignification, telescopic synthesis of cellulose was performed using 90% w/v of FA at 90 °C for 2 h. Subsequently, the catalytic oxidation reactions were evaluated at intervals over 24 h (see Fig. 1e). Similar to previous researches, at the extent of delignification was around 52.5% following organosolv extraction. High fractionation yields of lignin and cellulose have been reported by Zhang^40^ as well. Meanwhile, following catalytic oxidation, the extent of delignification was up to ~91.7% during the first 8 h of the reaction. From there, the efficiency of oxidation gradually decreased after 12 h, and by 24 h after the reaction only <0.5 wt% of insoluble lignin remained. Specifically, the cellulose yield from this integrated process was 30.1% of that of the raw EFB fibers.

### Cellulose nanofibrils

High-speed blending successfully defibrillated the cellulose within 30 min. Similar to our previous study, the resulting nanocellulose was clearly visible, and its stability was higher following 20–30 min of agitation^17^. Evidently, mechanical disintegration prevented the excessive degradation of cellulose^46,47^. During agitation, a control cycle was necessary because of the temperature rise to ~70 °C ten minutes following the commencement of agitation. Prolonged agitation could destroy and hydrolyze the fractional cellulose and reduce the yield. By maintaining the temperature of the defibrillation process, the CNF EFB yield relative to the initial weight of EFB fibers consistently 30.1% without loss of cellulose during defibrillation process (see Fig. 1b). Nevertheless, fiber losses during the subsequent processes (e.g. repetitive treatment, washing, etc.) could have been responsible for the differences in the total yields of the different processes.

### Chemical characterization of CNF EFB

**Figure 2:**
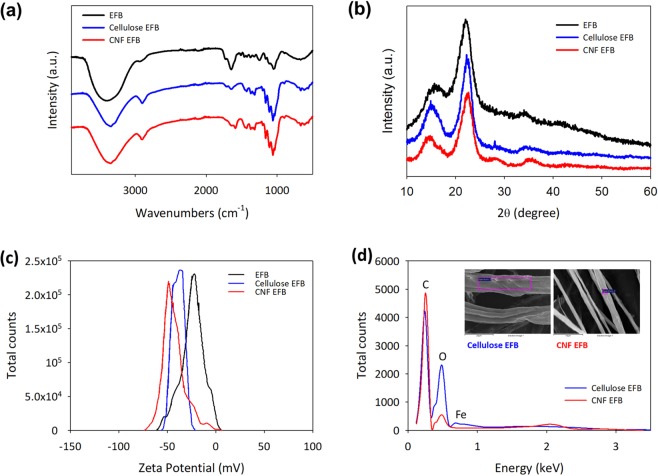
Chemical characterization of untreated EFB, isolated cellulose and CNF EFB on (**a**) FTIR, (**b**) XRD spectrum, (**c**) the zeta potentials and (**d**) VPSEM-EDS.

The functional groups of the untreated EFB fibers were largely accounted for by the typical components of lignocelluloses. Specifically, a broad intensity belonging to the hydroxyl groups were noted at 3693 to 3014 cm^−1^, while the carboxyl and carbonyl groups 1733 and 1 650 cm^−1^ respectively (see Fig. 2a). Moreover, the intensity of the carbonyl spectrum significantly changed post-cellulose isolation as a result of oxidation^11^. Additionally, lignin degradation via catalytic oxidation disrupted the guaiacyl and syringyl units of the lignin peaks at 1249 cm^−1^. Apparently, the differences in the functional groups between the cellulose isolation and cellulose defibrillation processes were negligible. As per Fig. 2b, the peak positions of the lignocellulosic component at the (002) and (004) planes were preserved during the telescopic processes. In general, degradation of non-cellulosic compositions (lignin, hemicellulose, etc.) and slight hydrolyzation of the amorphous regions greatly enhance the crystalline structure of cellulose^48^.

Even though the immense stability of the resulting nanocellulose could be optically determined by means of water suspension in the absence of particle sedimentation, additional information in the form of zeta potential provided in-depth interpretations of the stability of nanocellulosic suspensions. With reference to Fig. 2c, the zeta potentials of untreated EFB fibers, isolated cellulose, and synthesized CNF EFB were −24.1, −38.9, and −41.9 mV on average respectively. These results corresponded with previously-reported hypothetical zeta potentials of stable nanocellulose (i.e. less than −30 mV)^43,49,50^.

The cellulose and CNF EFB isolated from oil palm EFB were further analyzed via EDS in order to identify the presence of Fe(II) residues, especially after catalytic oxidation. In our previous study, electrostatic interactions between the positive charges of the cellulose (hydroxyl and carboxyl groups) freely adsorbed Fe(II) ions^16^. Accordingly, 0.06–0.27 wt% of Fe residues were present in the isolated cellulose and CNF EFB, as shown in Fig. 2d. The immense shearing forces during defibrillation have clearly disintegrated the cellulosic fraction and extracted most of the Fe residues from the preceding process^51^. Both cellulose and CNF EFB were mainly composed of carbon and oxygen. However, cellulose had a greater oxygen content than CNF EFB (38.4 and 26.8 wt%, respectively) as it was oxidized to a greater extent during catalytic oxidation^46^.

### The stability of CNF/Fe(0)

**Figure 3:**
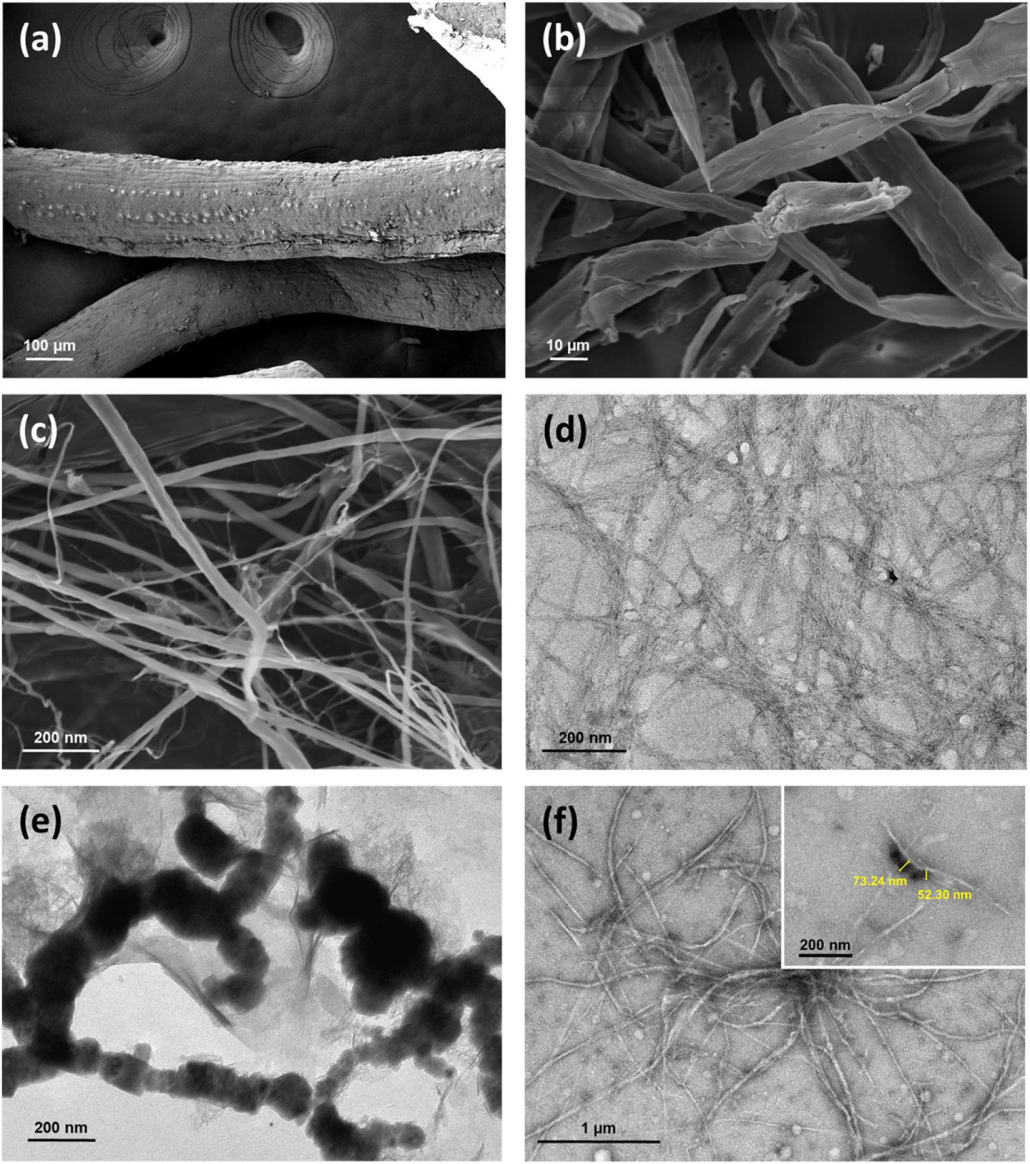
The morphological structure of (**a**) untreated EFB, (**b**) isolated cellulose, (**c**) CNF EFB and TEM images of (**d**) CNF EFB, (**e**) Fe(0) and (**f**) CNF/Fe(0).

The VPSEM micrograph in Fig. 3a shows the distinct morphological structures of the untreated EFB fibers following the telescopic processes of cellulose isolation and defibrillation. Evidently, the integrated process of organosolv lignin extraction and catalytic oxidation effectively fractionated the cellulosic fibers (Fig. 3b). Through these processes, the disruption of size and dislodgement of the silica bodies of the untreated EFB fibers became visible owing to the rougher surface of untreated EFB in comparison with cellulose fibers. In other words, mechanical treatment defibrillated the cellulosic fibers into a scattered and web-like structure (see Fig. 3c). Furthermore, the diameter of the CNF EFB (as per the TEM micrograph in Fig. 3d) was comparable with the previously-reported values which were in the range of 7.2–11.5 nm^17,18^.

**Figure 4:**
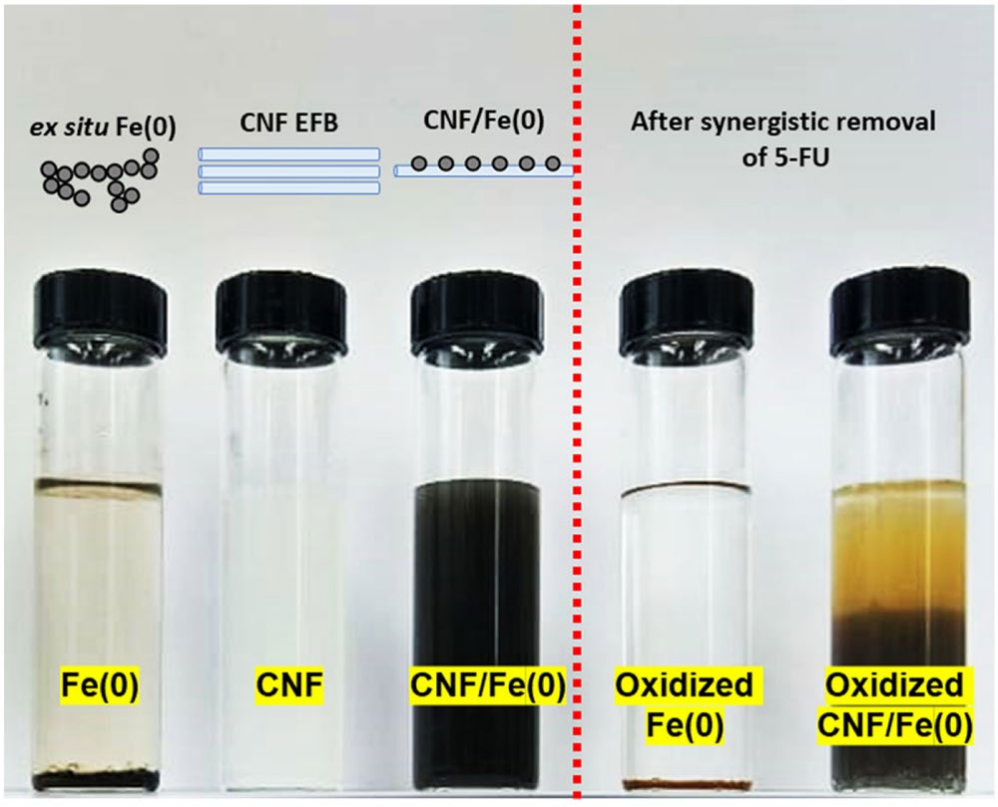
The formation and oxidation effects on *ex situ* Fe(0) and CNF/Fe(0) after the oxidation process.

Figure 3e shows the agglomerated and chain-like structures of the *ex situ* Fe(0) nanoparticles whose average diameters were 55.3–163.15 nm. In the absence of a stabilizing agent, Fe(0) nanoparticles are readily oxidized into iron oxide or hydroxide oxide^22^. On the contrary, the stable Fe(0) nanoparticles were well-distributed and -segregated in the suspension form of CNF EFB, whereby the diameters ranged from 52.3–73.24 nm (see Fig. 3f). The stability of CNF/Fe(0) was successfully take place and can be visually observed in water suspension (see Fig. 4). In the absence of CNF EFB, the colloidal Fe(0) particles was sedimented providing its instability and the formation of the agglomeration. Although the stability of the CNF/Fe(0) can be sustained for longer life cycle in comparison with *ex situ* Fe(0), however, the generation of sludge after the oxidation reactions still remains the limitation on the utilization of Fenton-like oxidation process.

**Figure 5:**
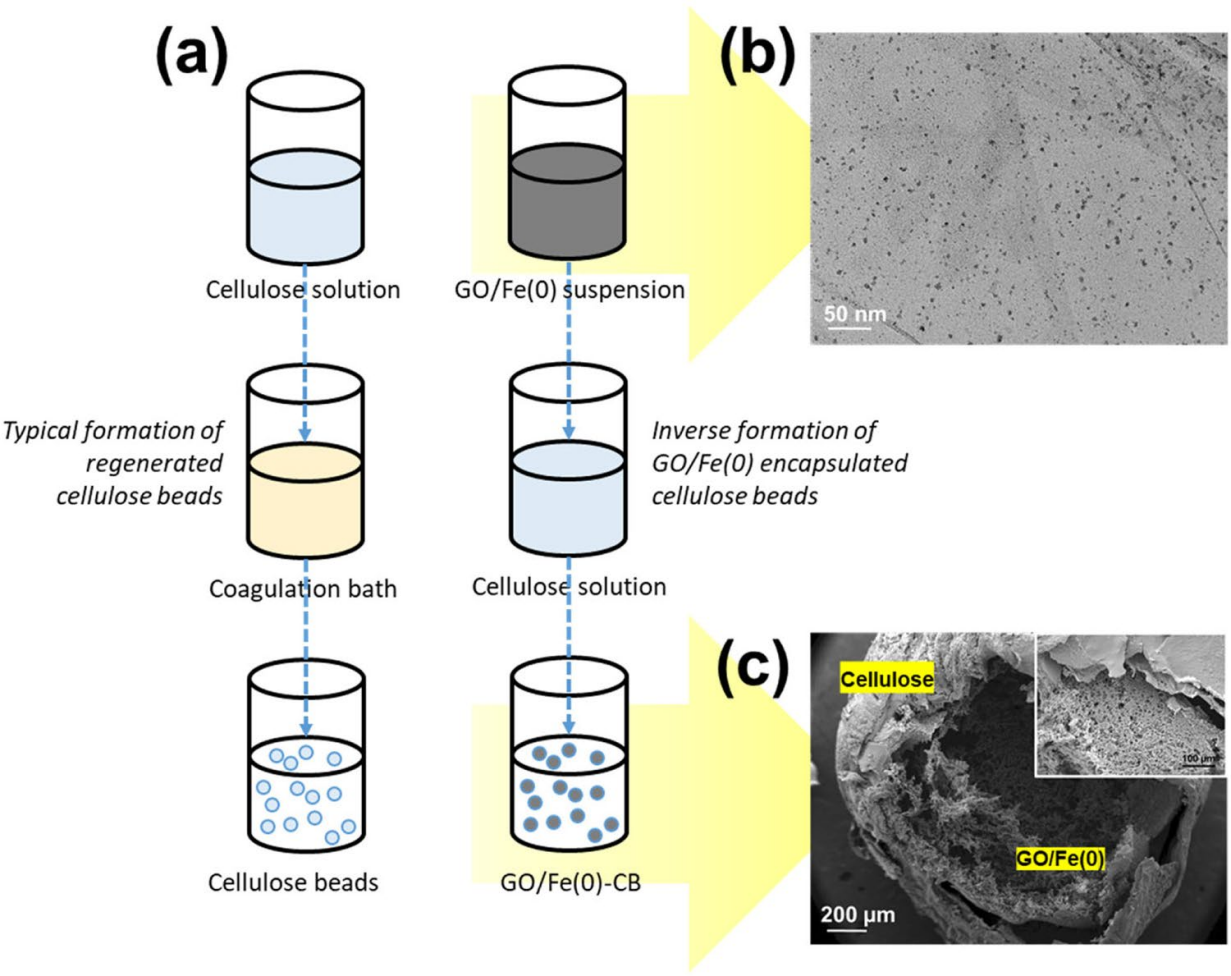
The inverse formation of the cellulose beads by encapsulating acidic suspension of GO/Fe(0) in a cellulose solution.

Alternatively, the FeOOH and Fe(OH)_3_ formation after the oxidation of Fenton’s reagent can still be managed by encapsulating Fe(0) particles in the regenerated cellulose beads. Typically, the formation of regenerated cellulose beads can be formed by continuously dropping cellulose solution through a syringe nozzle in the coagulation bath (5% H_2_SO_4_) shows in Fig. 5a. Contrary, the formation of encapsulated Fe(0) in the cellulose was done inversely, whereby GO solution is used as supporting material for Fe(0) particles (see Fig. 5b). Throughout the adsorption study, GO manage to uptake Fe(II) ions up to 18.38 mg/g. Whereas, the microstructure of regenerated cellulose and encapsulated GO/Fe(0) was observed thoroughly by VPSEM analysis (see Fig. 5c). The formation of cellulose beads encapsulated GO/Fe(0) exhibited both dense and porous structures at the inner and the outer layer of the hollow beads. The outer layer of the beads comprises with the layer of regenerated cellulose encapsulated the porous structure of GO/Fe(0). The regenerated cellulose shows a combination of low porosity and dense structure. While crosslinking effect between cellulose and GO through the intermolecular hydrogen bond has been constructed a three-dimensional network which contributed more porous structure^18,31^.

### Synergistic removal of 5-FU

**Figure 6:**
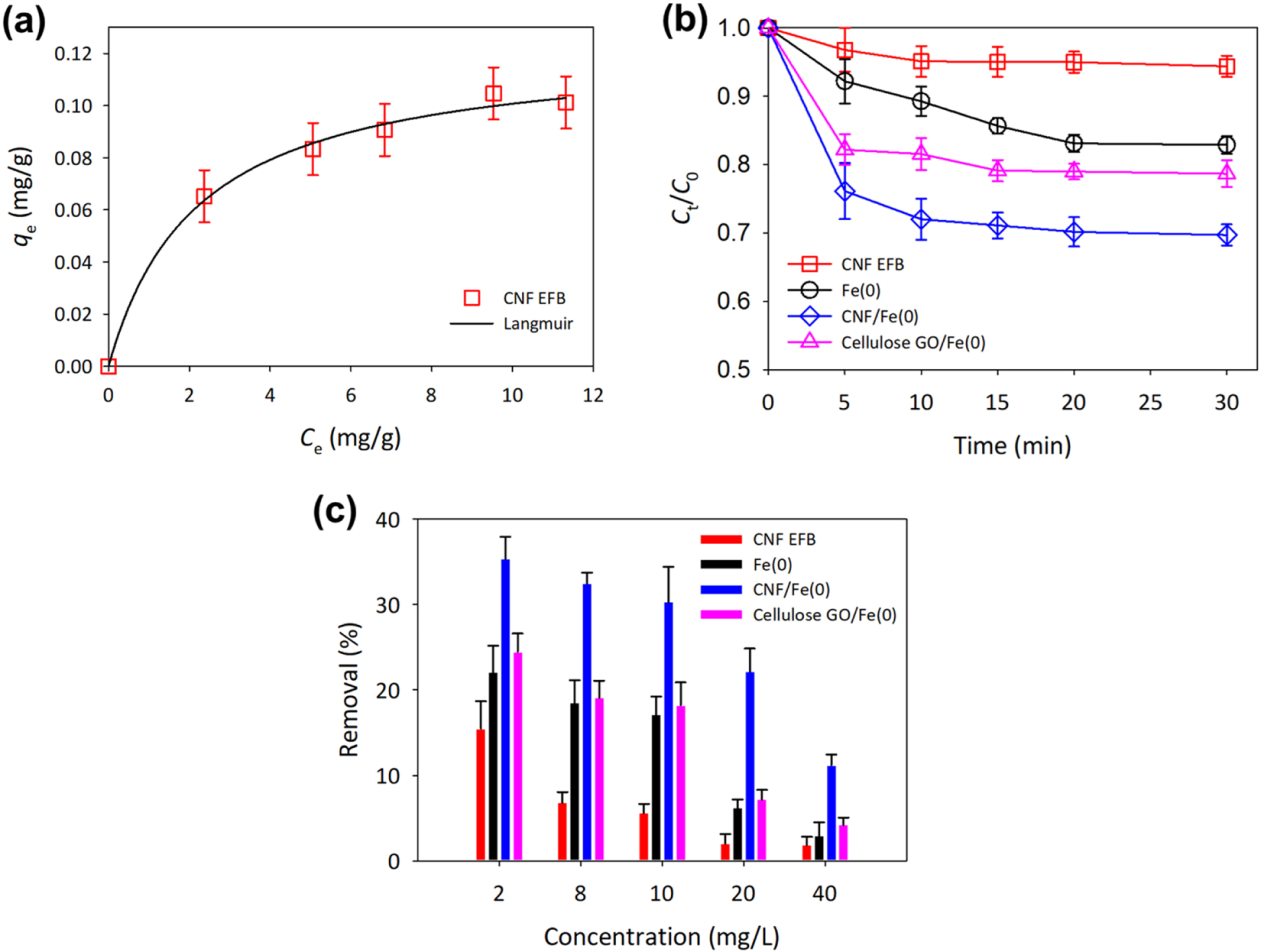
(**a**) The removal of 5-FU by CNF EFB in the fitting adsorption isotherm of Langmuir, (**b**) kinetics degradation of CNF EFB, *ex-situ* Fe(0), CNF/Fe(0) and cellulose GO/Fe(0) at 10 mg/L of 5-FU and (**c**) the efficiency of CNF EFB, *ex-situ* Fe(0), CNF/Fe(0) and cellulose GO/Fe(0) at different concentration of 5-FU.

The preliminary study on the adsorption performance of CNF EFB was conducted in different concentration of 5-FU (2–10 mg/L) as an estimation of the total 5-FU uptake and suggested mechanism interaction of CNF and 5-FU. The adsorption capacity of 5-FU on the CNF EFB has been computed using Langmuir isotherm expressed in Eq. (3)^52^;

3 $${q}_{e}=\frac{{Q}_{0}b{C}_{{\rm{e}}}}{{\rm{1}}+b{C}_{{\rm{e}}}}$$

where Langmuir isotherm described, *Q*_0_ is the maximum uptake of 5-FU per unit mass of adsorbent (mg/g) and *b* is a constant related to the adsorption energy (L/mg). The coefficient correlation, *r*^2^ between experimental and Langmuir predicted in Fig. 6a shows a high correlation at 0.994, which interprets the monolayer interaction of 5-FU on the CNF EFB. Given the negative charge of both CNF EFB and 5-FU, the interactions are expected to be weak, thus provided a low adsorption capacity. This was proven by the maximum adsorption capacity, *Q*_0_ calculated was 0.123 mg/g, which is comparatively lower than activated carbon^34^.

Meanwhile, the comparative performances of the adsorptive and oxidative techniques for the removal of 5-FU were evaluated at degradation kinetics of 10 mg/L of 5-FU for 30 min (see Fig. 6b). Evidently, the ability of CNF EFB to act as a rapid adsorbent was similar to those of prior studies as the adsorption equilibrium of 5-FU was attained within 10 min^18^. Also, the concentration of 5-FU gradually decreased during Fenton-like oxidation owing to the generation of •OH by the *ex situ* Fe(0) nanoparticles, the latter of which became saturated after 20 min of reaction^37^. The absent of hydrogen peroxide shows the possible reaction of Fe(0) as described in Eq. (4) to Eq. (6) below^53^.

4 $${{\rm{Fe}}}^{0}+{{\rm{O}}}_{2}+{{\rm{2H}}}^{+}\to {{\rm{Fe}}}^{2+}+{{\rm{H}}}_{2}{{\rm{O}}}_{2}$$

5 $${{\rm{Fe}}}^{0}+{{\rm{H}}}_{2}{{\rm{O}}}_{2}\to {{\rm{Fe}}}^{2+}+{{\rm{2OH}}}^{-}$$

6 $$\mathrm{Fe}(\mathrm{II})+{{\rm{H}}}_{2}{{\rm{O}}}_{2}\to {{\rm{Fe}}}^{3+}+{{\rm{OH}}}^{\bullet }+{{\rm{OH}}}^{-}$$

The reactivity of *ex situ* Fe(0) was initiated with the oxidation of Fe(0) by O_2_ for the formation of H_2_O_2_ and gradually produced •OH. The theoretical stoichiometry was proven by the previous study in the mineralization of dye and textile effluent^21,28^. Whereas, the transformation products generated from 5-FU (C_4_H_3_FN_2_O_2_) by Fenton oxidation are comprised of C_4_H_5_FN_2_O_4_ and C_4_H_7_NO_3_, which are partly involved by defluorination and dihydroxylation of C_4_H_3_FN_2_O_3_^54^.

Apparently, CNF EFB and *ex situ* Fe(0) alone only managed to remove 1.87 and 2.93% of 40 mg/L 5-FU respectively (see Fig. 6c). The integration between CNF EFB and Fe(0) nanoparticles was further improved by the synergistic removal of 5-FU via adsorptive and oxidative techniques. The removal performance of CNF/Fe(0) was way better than either of them alone; even when the concentration of 5-FU was at its highest, 11.2% of the same got removed. Alternatively, this synergistic performance also provided a similar trend by encapsulated GO/Fe(0). However, without the maximum exposure of Fe(0) nanoparticles inside cellulose beads, the capability to remove 5-FU was distinctly reduced.

Apart from the synergistic effect of adsorption-oxidation, the formation of stable Fe(0) nanoparticles was partially attributable to the increased removal of 5-FU. Unlike *ex situ* Fe(0) nanoparticles generated by colloidal formation, CNF/Fe(0) had better stability and greater homogeneous suspension in water^38^ (see Fig. 4). The deposited Fe(0) nanoparticles could be sustained even when the Fe(0) load on the CNF EFB was 10 wt%. In terms of the mobility of Fe(0) nanoparticles, the reactivity of the colloidal Fe(0) with 5-FU was likely to be restricted^22^. Despite the absence of interactions with 5-FU, Fe(0) will still be gradually oxidized into iron oxides. On the contrary, the encapsulated GO/Fe(0) was able to emulate the efficiency of *ex situ* Fe(0) nanoparticles at the range of the concentration tested. As a result of the GO capability in uptaking Fe(II) ions, the residue from Fenton-like oxidation was able to contain inside of encapsulated GO/Fe(0) cellulose beads.

## Conclusions

This study has proposed a method to telescopically synthesize cellulose to cellulose nanofibrils from oil palm EFB fibers via controlled processes of organosolv extraction, catalytic oxidation, and mechanical defibrillation. This method has significantly improved the yields of cellulose and cellulose nanofibrils without compromise in quality. Furthermore, since the excess Fe(II) ions following cellulose preparation were still intact, *in situ* formation of Fe(0) was viable, and its stability was better than those which were generated *ex situ*. The combination of CNF and Fe(0) facilitated the removal of high concentrations of 5-FU as well. Besides, encapsulating GO/Fe(0) in cellulose beads offering alternative approaches in the integration of adsorption-oxidation at manageable sludge residues.

## References

[1] Malaysian Palm Oil Board (MPOB) (2018) Overview of the Malaysian oil palm industry 2017, http://palmoilis.mpob.gov.my/index.php/overview-of-industry/593-overview-of-industry-2017. Accessed 10 December 2018.

[2] Palamae S, Dechatiwongse P, Choorit W, Chisti Y, Prasertsan P (2017). Cellulose and hemicellulose recovery from oil palm empty fruit bunch (EFB) fibers and production of sugars from the fibers. Carbohyd Polym.

[3] Carlmark A, Larsson E, Malmström E (2012). Grafting of cellulose by ring-opening polymerisation – A review. Eur. Polym. J..

[4] Kaco H, Zakaria S, Chia CH, Zhang L (2014). Transparent and printable regenerated kenaf cellulose/PVA film. BioResources.

[5] Chaturvedi V, Verma P (2013). An overview of key pretreatment processes employed for bioconversion of lignocellulosic biomass into biofuels and value added products. 3 Biotech.

[6] Tadesse H, Luque R (2013). Advances on biomass pretreatment using ionic liquids: an overview. Energy Environ. Sci..

[7] Upton BM, Kasko AM (2016). Strategies for the conversion of lignin to high-value polymeric materials: review and perspective. Chem. Rev..

[8] Li MF (2017). Sequential two-step fractionation of lignocellulose with formic acid organosolv followed by alkaline hydrogen peroxide under mild conditions to prepare easily saccharified cellulose and value-added lignin. Energy Convers. Manage..

[9] Kumar P, Barret DM, Delwiche MJ, Stroeve P (2009). Methods for pretreatment of lignocellulosic biomass for efficient hydrolysis and biofuel production. Ind. Eng. Chem. Res..

[10] Marques G (2010). Delignification of eucalypt kraft pulp with manganese-substituted polyoxometalate assisted by fungal versatile peroxidase. Bioresource Technol..

[11] Collinson SR, Thielemans W (2010). The catalytic oxidation of biomass to new materials focusing on starch, cellulose and lignin. Coordin. Chem. Rev..

[12] Sugano Y (2016). specific electrocatalytic oxidation of cellulose at carbon electrodes modified by gold nanoparticles. ChemCatChem.

[13] Gaspar AR, Gamelas JAF, Evtuguin DV, Neto CP (2007). Alternatives for lignocellulosic pulp delignification using polyoxometalates and oxygen: a review. Green Chem..

[14] Cao JH, Zhao JR (2015). Fenton depolymerization of cellulosic biomass in modified cuprammonium solution. BioResources.

[15] Arantes V, Milagres AMF (2006). Degradation of cellulosic and hemicellulosic substrates using a chelator-mediated Fenton reaction. J. Chem. Technol. Biot..

[16] Santanaraj J (2017). Enhance delignification of oil palm empty fruit bunch fibers with *in situ* fenton-oxidation. Bioresources.

[17] Chan CH, Chia CH, Zakaria S, Sajab MS, Chin SX (2015). Cellulose nanofibrils: a rapid adsorbent for the removal of methylene blue. RSC Adv..

[18] Sajab MS (2016). Bifunctional graphene oxide–cellulose nanofibril aerogel loaded with Fe (III) for the removal of cationic dye via simultaneous adsorption and Fenton oxidation. RSC Adv..

[19] Sun YP, Li XQ, Cao J, Zhang WX, Wang HP (2006). Characterization of zero-valent iron nanoparticles. Adv. Colloid. Interfac..

[20] Pereira WS, Freire RS (2006). Azo dye degradation by recycled waste zero-valent iron powder. J. Braz. Chem. Soc..

[21] Sajab MS (2019). Insight observation into rapid discoloration of batik textile effluent by *in situ* formations of zero valent iron. Sains Malays..

[22] Feitz JA (2005). Oxidative transformation of contaminants using colloidal zero-valent iron. Colloid. Surface A.

[23] Phenrat T, Saleh N (2007). Aggregation and sedimentation of aqueous nanoscale zerovalent iron dispersions. Environ. Sci. Technol..

[24] Rosická D, Sembera J (2011). Influence of structure of iron nanoparticles in aggregates on their magnetic properties. Nanoscale. Res. Lett..

[25] Duarte F, Morais V, Maldonado-Hódar FJ, Madeira LM (2013). Treatment of textile effluents by the heterogeneous Fenton process in a continuous packed-bed reactor using Fe/activated carbon as catalyst. Chem. Eng. J..

[26] Lu Q (2019). *In situ* synthesis of a stable Fe3O4@cellulose nanocomposite for efficient catalytic degradation of methylene blue. Nanomaterials.

[27] Santos FSD, Lago FR, Yokoyama L, Fonseca FV (2017). Synthesis and characterization of zero-valent iron nanoparticles supported on SBA-15. J. Mater. Res. Technol..

[28] Yoo SH, Jang D, Joh HI, Lee S (2017). Iron oxide/porous carbon as a heterogeneous Fenton catalyst for fast decomposition of hydrogen peroxide and efficient removal of methylene blue. J. Mater. Chem. A.

[29] He Y, Jiang DB, Jiang DY, Chen J, Zhang YX (2018). Evaluation of MnO2-templated iron oxide-coated diatomites for their catalytic performance in heterogeneous photo Fenton-like system. J. Hazard. Mater..

[30] Sharma AK (2018). Reductive-co-precipitated cellulose immobilized zerovalent iron nanoparticles in ionic liquid/water for Cr(VI) adsorption. Cellulose.

[31] Yao L, Lu Y, Wang Y, Hu L (2014). Effect of graphene oxide on the solution rheology and the film structure and properties of cellulose carbamate. Carbon.

[32] Mahnik SN, Lenz K, Weissenbacher N, Mader RM, Fuerhacker M (2007). Fate of 5-fluorouracil, doxorubicin, epirubicin, and daunorubicin in hospital wastewater and their elimination by activated sludge and treatment in a membrane-bio-reactor system. Chemosphere.

[33] Tsakona E, Anagnostopoulou M, Gidarakos E (2007). Hospital waste management and toxicity evaluation: A case study. Waste Manage..

[34] Kovalova L, Knappe DRU, Lehnberg K, Kazner C, Hollender J (2013). Removal of highly polar micropollutants from wastewater by powdered activated carbon. Environ. Sci. Pollut. Res..

[35] Jiang G (2017). Identification of active hydrogen species on palladium nanoparticles for an enhanced electrocatalytic hydrodechlorination of 2, 4-dichlorophenol in water. Environ. Sci. & Technol..

[36] Jiang G (2018). Electrocatalytic hydrodechlorination of 2,4-dichlorophenol over palladium nanoparticles and its pH-mediated tug-of-war with hydrogen evolution. Chem. Eng. J..

[37] Gazenko O (2017). Fast and complete removal of the 5-fluorouracil drug from water by electro-Fenton oxidation. Environ. Chem. Lett..

[38] Mirzaei A, Chen Z, Haghighat F, Yerushalmi L (2017). Removal of pharmaceuticals from water by homo/heterogonous Fenton-type processes a review. Chemosphere.

[39] Hummers WS, Offeman RE (1958). Preparation of grahitic oxide. J. Am. Chem. Soc..

[40] Zhang Y (2018). One-step fractionation of the main components of bamboo by formic acid-based organosolv process under pressure. J. Wood Chem. Technol..

[41] Kunaver M, Anžlovar A, Žagar E (2016). The fast and effective isolation of nanocellulose from selected cellulosic feedstocks. Carbohyd. Polym..

[42] Zhang J (2010). Isolation and characterization of wheat straw lignin with a formic acid process. Bioresource Technol..

[43] Mohaiyiddin MS (2016). Characterization of nanocellulose recovery from Elaeis guineensis frond for sustainable development. Clean Technol. Environ..

[44] Dai J, Patti AF, Saito K (2016). Recent developments in chemical degradation of lignin: catalytic oxidation and ionic liquids. Tetrahedron Lett..

[45] Hellström P, Heijnesson-Hultén A, Paulsson M, Håkansson H, Germgård U (2014). The effect of Fenton chemistry on the properties of microfibrillated cellulose. Cellulose.

[46] Nechyporchuk O, Belgacem MN, Bras J (2016). Production of cellulose nanofibrils: A review of recent advances. Ind. Crop. Prod..

[47] Jonoobi M (2015). Different preparation methods and properties of nanostructured cellulose from various natural resources and residues: a review. Cellulose.

[48] Deepa B (2015). Utilization of various lignocellulosic biomass for the production of nanocellulose: a comparative study. Cellulose.

[49] Faradilla RHF (2016). Nanocellulose characteristics from the inner and outer layer of banana pseudo-stem prepared by TEMPO-mediated oxidation. Cellulose.

[50] Filipova I, Fridrihsone V, Cabulis U, Berzins A (2018). Synthesis of nanofibrillated cellulose by combined ammonium persulphate treatment with ultrasound and mechanical processing. Nanomaterials.

[51] Naduparambath S (2018). Isolation and characterisation of cellulose nanocrystals from sago seed shells. Carbohyd. Polym..

[52] Langmuir I (1916). The constitution and fundamental properties of solids and liquids. Part. I: Solids. J. Am. Chem. Soc..

[53] Joo SH, Feitz AJ, Sedlak DL, Waite TD (2005). Quantification of the oxidizing capacity of nanoparticulate zero-valent iron. Environ. Sci. Technol..

[54] Lutterbeck CA (2015). Degradation of 5-FU by means of advanced (photo) oxidation processes: UV/H2O2, UV/Fe2+/H2O2 and UV/TiO2—comparison of transformation products, ready biodegradability and toxicity. Sci. Total Environ..

